# What is attractive rural landscape? Differences in the social and expert assessment of the changes in the rural landscape of the Carpathian region in Poland with regard to the need of its protection

**DOI:** 10.1007/s11629-022-7377-7

**Published:** 2023-02-01

**Authors:** Agata Gajdek, Barbara Krupa, Anna Nowak

**Affiliations:** Department of Landscape Architecture, Institute of Agricultural Sciences, Environmental Protection and Development, College of Natural Sciences, University of Rzeszow, ul. Ćwiklińskiej 2, PL-35-601 Rzeszów, Poland

**Keywords:** Polish village, Landscape attractiveness, Cultural landscape, Spatial systems

## Abstract

Contemporary villages of the mountain region are subject to uncontrolled structural and spatial transformations, which cause deformation of centuries-old spatial systems of high cultural and natural value. The aim of the study is to confront the opinions of the inhabitants and experts regarding the condition of the cultural landscape of the villages in south-eastern Poland. This area belongs to the Carpathian region of Central Europe. The historical and economic conditions of the studied region, related to the functioning in the post-war period, and then its breakdown and the development of the free market economy, constitute an interesting background for the proposed research. Local communities still remembering the period of difficulties related to the period of systemic transformations, are currently experiencing a relative prosperity, many difficulties related to the period of systemic transformations, are currently experiencing a relative prosperity, which is also expressed in a completely new, previously unknown way of managing the landscape. Investments implemented in villages are associated by the inhabitants with the improvement of the standards and quality of life. They assess them rather positively. An expert assessment of these landscape transformations indicates their negative dimension and the risk of losing timeless values. The discrepancy in the assessment of experts and local residents creates difficulties in the protection of the rural landscape. Therefore, high-quality visual landscape features among rural residents is necessary from the point of view of its multi-faceted and effective protection. Local initiatives and actions in the field of industry policy should play a significant role in this respect by consolidating the images of a harmonious landscape in the public awareness.

## 1 Introduction

Shaping the landscape of rural areas has the multifaceted character and is the subject of considerations of numerous scientists (Palang et al. 2004; Modica & Di Fazio 2018). The European continent has rich rural landscape heritage, albeit with varying degrees of integrity and preservation (Agnoletti 2012; Sánchez et al. 2020). The pace and scope of cultural, technological and economic changes that have occurred in recent decades threaten not only the environment, but also the related rural landscapes and communities (Antrop 2006; Torquati et al. 2017). The uncontrolled degradation of the rural landscape has also been taking place in Poland. The lack of decisive actions aimed at defining clear rules of conduct leads to the loss of regional diversity (Raszeja 2013; Belof 2013). Contemporary threats to the values of the landscape and cultural heritage of villages include: adopting urban architectural patterns, dispersion of the housing structures, the presence of large-scale and high-rise buildings, lack of protection of scenic views and passive exposure of panoramic zones (showing the silhouette of the village in the landscape), the disappearance of traditional elements of the agricultural landscape, as well as deformation and the disappearance of traditional home gardens (Raszeja 2013). These phenomena result to a significant extent from widespread globalization, leading to landscape unification (Chmielewski 2013).

For decades, rural landscapes have undergone rapid and sometimes radical changes in different directions. The same multiple changing trends on different scales are observed in many regions of the world (Pinto-Correiaa & Kristensenb 2013). Thorbeck (2012) suggests that in the context of spatial planning for rural areas, a completely new approach should be developed that would take into account the transformations of these areas. The measures taken so far to deal with the multidimensional crisis of rural areas led to the "urbanization" of rural areas and the industrialization of agriculture, which resulted in the disappearance of their individuality and identity, and thus degradation of the rural cultural landscape. The answer to civilization changes was the idea of renewal, consisting in the search for development concepts in line with the natural, social, economic and cultural specificity of rural areas, based on local resources and with the participation of local communities (Wilczyński 2003; Tieskens et al. 2018).

Additionally, the laws in force in Poland are not effective in properly shaping the rural landscape. Activities in rural areas should be related to the communities that live and function in this area and have a significant impact on its shaping (Pawłowska 2014; Kuriata et al. 2014; Wartmann et al. 2021). The rural landscape treated as a historical product demands to be properly assessed with identification of its threats and critical reference to political directives and research. The approaches developed in recent decades have a negative impact on the protection and management of landscape values. The landscape directives strengthened the trend towards cultural globalization, encouraging renaturalization, especially in terms of forestation, neglecting ancient landscape patterns and long, rich cultural history that resulted in their creation (Agnoletti 2014).

Finally, sometimes the inhabitants of the countryside themselves contribute to the disappearance of the legible character of rural areas (Kowicki 2010). The study on the perception of landscape by inhabitants has shown that the way landscape has been perceived evolves with the place and identity of people (Manzo 2003; Almeida et al. 2016). Demographic and functional changes in landscape influence its perception by the inhabitants (DeLucio & Mugica 1994) and the perception of this landscape (Kaplan 1985b). Coeterier (1996) exacerbates this by saying that people increasingly pay attention to the quality of the landscape and consider its preservation to be an important aspect. The landscape should be well-kept and ordered (Rogge et al. 2007; Bella et al. 2018). Scott (2002) points out that "ordinary citizens" perceive the landscape as whole, they rarely divide it into component parts. "Experts", on the other hand, tend to bypass local knowledge of a place (Roberts 1998). Strumse (1994b, 1996) states that lay people generally rate agricultural landscapes higher than experts. Taking into account the assessment and changes of the landscape, the inhabitants seem to see it as constantly changing. People who are not emotionally connected with the assessed place treat the landscape as a static view and have a clear idea of what it should look like (Soini et al. 2012). Changes in the rural landscape are perceived differently by the inhabitants of rural areas and by the visitors who have different expectations as to what the countryside should be like, how and what for the landscape should be used (Meeus & Gulinck 2008; Boğaç 2020).

The existence of discrepancies in the assessment of landscape changes by various social groups has already been studied and described by the representatives of various scientific disciplines. However, we were interested in confronting the opinions of rural residents with the opinions of experts – landscape architects. The historical context of the study area is important, in which rural origin was perceived negatively and during the period of systemic transformations it was used for propaganda and political purposes. “Being from the countryside” was synonymous to “being inferior” at that time. The current modernizations of Polish rural areas visually bring the village closer to the urban housing estate (Deslatte et al. 2022), which takes place with the social consent of the inhabitants. In addition, areas similar to our study generally do not have a landscape policy. When planning our research, we anticipated that despite such unambiguous transformations, the evaluation of the landscape attractiveness of these villages by the inhabitants would be rather high. A renovated village, a developing village, a village equipped with all amenities, is an attractive village, also with an attractive landscape. The research thesis was formulated in this way: the lack of a long-term policy of shaping the landscape of the surveyed towns does not lower the assessment of their attractiveness by the inhabitants, if an interesting (though not always adequate to local conditions) way of their arrangement and aesthetics is ensured.

## 2 Characteristics of the Rural Landscape of South-Eastern Poland

The cultural and historical heritage of the Carpathians is one of the main distinguishing features of this region in Europe and the world. The awareness of the richness of the common cultural heritage strengthens the sense of identity and dignity of the inhabitants of the Carpathian region.

Over the centuries, the Carpathian culture has been shaped by numerous ethnic groups, ranging from the Podhale highlanders, together with i.e. Highlanders of Wisła, Jabłonki, Babia Góra, Żywiec, Spiš, Pieniny, Sącz (in the western part of the Polish Carpathians), and Szlachtowa Rusyn, Eastern and Western Foothill inhabitants, Dolinians, Lemko and Bojko (in the eastern part) (Laskowicz & Mrozek 2019).

In addition, the cultural heritage of the Carpathian region is a common achievement of groups living not only in the Polish part of the Carpathians, but also in areas currently located within the borders of the Czechia, Romania, Slovakia, Serbia, Ukraine and Hungary. Its richness and diversity result primarily from the centuries-old coexistence, interaction and cultural exchanges of many nations living in the Carpathian Mountains, as well as local ethnic groups. The evolution of the cultural heritage of the Carpathians was also influenced by various external factors, including migrations and settlement in the Carpathians between the 13th and 17th centuries of the Wallachian population or the impact of many different religious denominations of the Western and Eastern rites (Laskowicz & Mrozek 2019).

The villages located in the Carpathian region are characterized by an attractive landscape location – river valleys, arable fields and woodland areas (Table 1).

All the studied localities are located in the Podkarpackie Province. The Podkarpackie Province is located in south-east of Poland, in the Carpathian region, the Western Carpathians province with the Western and Northern Subcarpathian (Polish physical and geographic regions according to J. Kondracki). To the north and west, it borders on the Małopolskie, Świętokrzyskie and Lubelskie provinces; to the south with Slovakia and to the east with Ukraine (Fig. 1). It is administratively divided into 25 poviats and 160 communes. The province covers the area of 17,846 km² (5.7% of the country area), inhabited by over 2.1 million inhabitants (5.5% of the country population). The average population density is 119 people per km² and is lower than the national average of 123 people per km² (Stanny et al. 2018).

The capital of the province is the city of Rzeszów, from which the distance of the studied villages is as follows: Zarzecze – 10.5 km, Wydrze – 26.6 km, Bachórz – 30 km, Chałupki – 38.2 km, Kuryłówka – 44.3 km, Wojtkowa – 65.6 km (Fig. 1). The surveyed localities are located very close (up to 5 km) or close (up to 20 km) to a small urban center with a population up to 15,000 (Fig. 1).

The studied villages are characterized by a stable demographic situation in the years 1998-2018. As a matter of fact some of them experienced a demographic decline, but it was insignificant, ranging from 0.2 to 3.9%. On the other hand, in the remaining localities, a demographic increase of 9.9% was observed (Fig. 1). This trend was in line with the demographic situation of the province, where the number of inhabitants in this period increased by 0.3% (polskawliczbach.pl).

**Table 1** The year of foundation and the historical and contemporary plan of the studied localities. Developed by the authors based on mapire.eu and geoportal.gov.pl

| Village (The first note of the locality) | XIX-century village plan | Contemporary village plan |
|---|---|---|
| Bachórz 1436 |  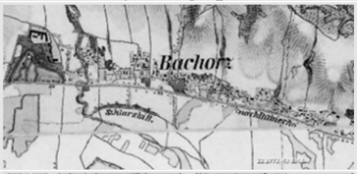 |  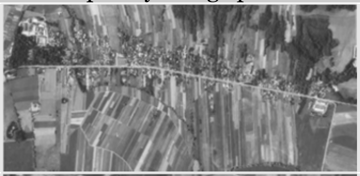 |
| Chałupki 1650 |   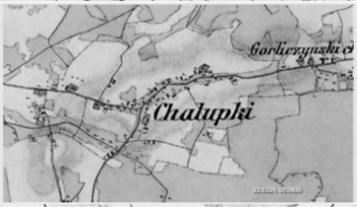 |  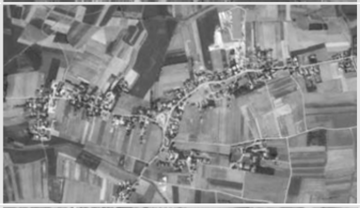 |
| Kuryłówka 1589 |   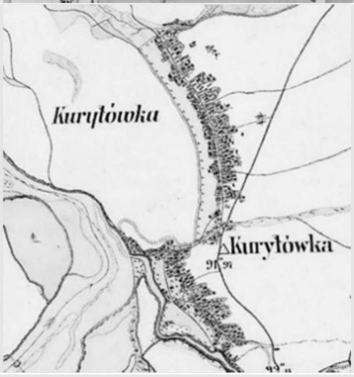 |  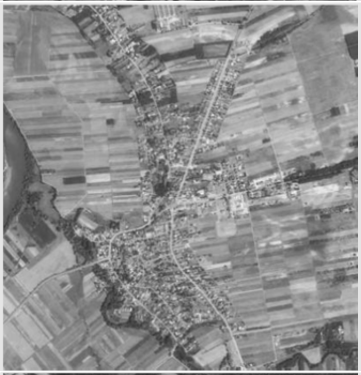 |
| Wojtkowa 1473 |   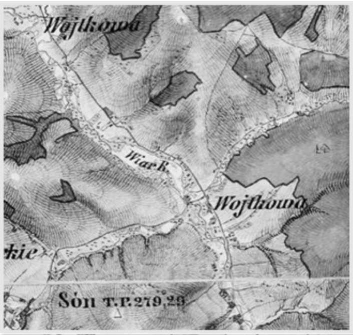 |  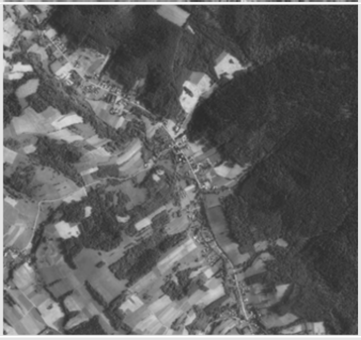 |
| Wydrze 1695 |  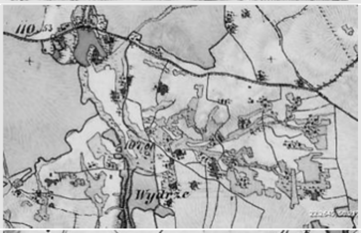 |  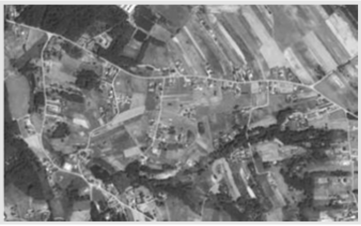 |
| Zarzecze 1423 |  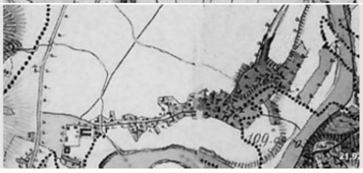 |  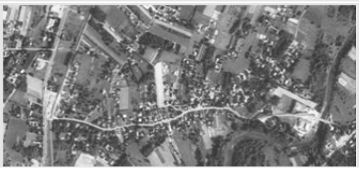 |

## 3 Methodology

### 3.1 Methodology of social assessment

In order to get acquainted with the social opinion of the attractiveness of the rural landscape, we constructed a questionnaire initially consisting of a dozen or so closed questions. The conducted pilot study on a group of 30 people allowed to verify the structure of the survey and make some modifications.

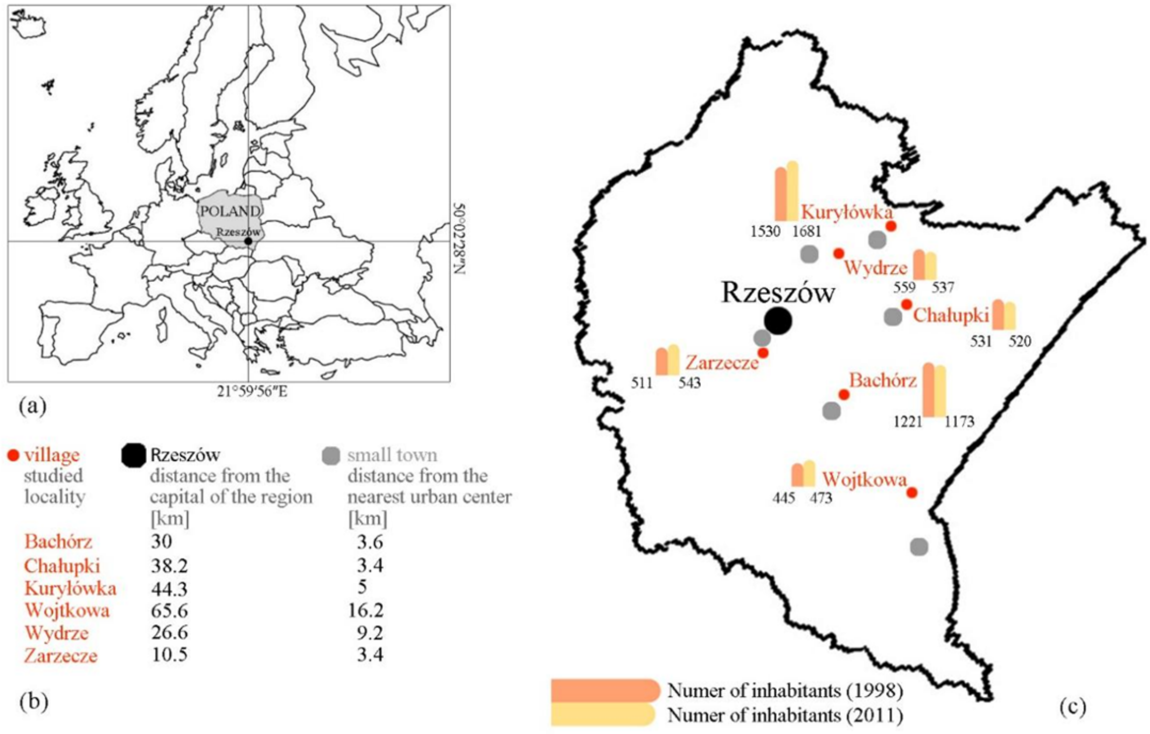

**Fig. 1** Study area. (a) Location of the study area in Europe and Poland; (b) Distance of the examined localities from the capital of the province (Rzeszów) and the nearest urban centers (towns with a population of less than 15,000); (c) Studied villages: Kuryłówka (1: 50°17′50″N 22°27′53″E), Wydrze (2: 50°12′53″N 22°15′34″E), Chałupki (3: 50°05′03″N 22°31′37″E), Zarzecze (4: 50°31′38″N 22°11′58″E), Bachórz (5: 49°50′13″N 22°16′08″E), Wojtkowa (6: 49°34′15″N 22°33′15″E). Location of the examined localities from the capital of the province (Rzeszów) and the nearest urban centers. Demographic changes in the examined villages in 1998–2011.

**Table 2** List of survey questionnaires.

| Question number | Questions |
|---|---|
| 1 | Do you consider the place of your residence as attractive in terms of landscape? |
| 2 | Has the landscape in your locality changed over the last 20 years (new buildings, road investments, landscaped green areas, renewable energy sources, landscape degradation? |
| 3 | What changes have been made to the landscape in your locality over the last 20 years? |
| 4 | Do you positively evaluate the changes in the landscape of your locality that have occurred over the last 20 years? |
| 5 | Do you think that the decisions of the commune concerning spatial solutions take into account the spatial order, harmony and beauty of the landscape in the place where you live? |
| 6 | What elements threaten the quality of the landscape in your locality? |

Ultimately, the questionnaire consisted of demographics and 6 basic questions (Table 2). On the one hand, the questions were to illustrate whether the inhabitants are observing spatial changes in their localities (especially in the last 20 years), and if so, what kind of changes are these and how they are assessed by the local community. We conducted the survey in 2021, in the period from March 30 to June 1. The diagnosis covered the 315 inhabitants of six villages Podkarpackie (Wojtkowa, Wydrze, Karyłówka, Zarzecze, Chałupki, Bachórz). Each of the respondents completed it on their own (Table 3).

Statistical analysis of the collected material was carried out in the Statistica 13.3 package by StatSoft. Qualitative data was analyzed. The relationships between the variables were assessed with the Pearson chi-square test ($\chi^2$). The results are presented in the form of frequencies, numbers and percentages, in multi-way tables. The level of statistical significance was $p < 0.05$.

## 3.2 Methodology of expert assessment

The expert assessment was carried out on the

**Table 3** Information about 315 respondents participated in the questionnaire survey.

| Items | | Data |
|---|---|---|
| Sampling population percentage in different villages | Wydrze | 19.0% |
| | Bachórz | 16.2% |
| | Zarzecze | 16.5% |
| | Wojtkowa | 15.9% |
| | Chałupki | 15.9% |
| | Kuryłówka | 16.5% |
| Sex | Woman | 52.7% |
| | Man | 47.3% |
| Age | 17-24 years old | 31.1% |
| | 25-34 years old | 25.4% |
| | >35-44 years old | 16.5% |
| | >45-54 years old | 13.7% |
| | 55-67 years old | 8.9% |
| | over 67 years old | 4.4% |
| Education | primary education | 15.6% |
| | secondary education | 57.8% |
| | higher education | 26.7% |

basis of field visions and the GIS environmental analyses, which were helpful in the development of analysis of the variability of the spatial structure of the studied landscape at the beginning of the 21th century based on the CORINE Land Cover (CLC) data from 2000 and 2018, the changes that took place within a radius of 2.5 km around the surveyed towns were examined. The analysis included the calculation of the area (ha) of selected types of use and the percentage of increase or decrease in the analysed period. The CLC 2000 database has been established as a data source for document preparation at national and regional level. The CLC program is run by the EEA. Its primary purpose is to document changes in land cover (Bossard et al. 2000; Büttner et al. 2004). The program collects data on land use on three levels. The first level covers the five main possible types of cover (artificial surfaces, agricultural areas, forest and semi-natural ecosystems, wetlands, water areas), the second level has 15 land cover forms, and the third level identifies 44 classes.

## 4 Results

The way in which the respondents perceived their place of residence in terms of landscape attractiveness was not homogenous (Table 4). The village of Bachórz was considered the least attractive in terms of landscape. Other towns were assessed similarly in this respect. The landscape values of Kuryłówka were assessed poorer than the remaining localities, while Wojtkowa was assessed the best.

The respondents also differently assessed own localities in terms of their landscape changing over the last twenty years (Table 5). The inhabitants of Bachórz were the least likely to notice landscape changes in their place of residence. The frequency of observation of changes by the inhabitants of other localities was similar, although the changes were most often noticed by the inhabitants of Kuryłówka.

Intensive urbanization processes and the disappearance of agricultural functions in rural areas or wind farms were among the rarest landscape changes observed, and their frequency did not differ depending on the locality studied ($p$>0.05). The

**Table 4** Inhabitants' perception of their places of residence as attractive in terms of landscape.

| Perception | Wydrza | Bachórz | Zarzecze | Wojtkowa | Chałupki | Kuryłówka | Total |
|---|---|---|---|---|---|---|---|
| Definitely yes | 48.3% | 5.9% | 42.3% | 52.0% | 40.0% | 46.2% | 39.4% |
| Rather yes | 48.3% | 58.8% | 55.8% | 40.0% | 48.0% | 36.5% | 47.9% |
| Rather no | 0.0% | 19.6% | 0.0% | 6.0% | 0.0% | 5.8% | 5.1% |
| Definitely no | 1.7% | 11.8% | 0.0% | 2.0% | 0.0% | 0.0% | 2.5% |
| I don't know/ hard to say | 1.7% | 3.9% | 1.9% | 0.0% | 12.0% | 11.5% | 5.1% |
| Total | 100.0% | 100.0% | 100.0% | 100.0% | 100.0% | 100.0% | 100.0% |
| $p$ | $\chi^2(20)=87.34$, $p<0.001$ <br> $\chi^2NW(20)=91.28$, $p<0.001$ | | | | | | |

**Table 5** Inhabitants' perception of the landscape of their places of residence as changing over the last twenty years (new buildings, road investments, landscaped green areas, renewable energy sources, landscape degradation).

| Perception | Wydrza | Bachórz | Zarzecze | Wojtkowa | Chałupki | Kuryłówka | Total |
|---|---|---|---|---|---|---|---|
| Yes | 91.7% | 68.6% | 94.2% | 94.0% | 94.0% | 96.2% | 90.2% |
| No | 3.3% | 17.7% | 3.9% | 6.0% | 0.0% | 3.9% | 5.7% |
| I don't know/ hard to say | 5.0% | 13.7% | 1.9% | 0.0% | 4.0% | 0.0% | 4.1% |
| Total | 100.0% | 100.0% | 100.0% | 100.0% | 100.0% | 100.0% | 100.0% |
| $p$ | $\chi^2(10)=36.33$, $p<0.001$ <br> $\chi^2NW(10)=35,03$, $p<0.001$ | | | | | | |

frequency of indicating other landscape changes was not consistent in six localities ($p$<0.05).

The new housing areas were most often mentioned by the inhabitants of Zarzecze, and the least frequently by the inhabitants of Wojtkowa. The construction of new local roads was definitely most often indicated by the inhabitants of Wojtkowa compared to other localities. The inhabitants of Wydrze, Bachórz and Kuryłówka mentioned a marked decrease in the area of green areas. A clear increase in the area of arranged green areas was observed most often by the inhabitants of Bachórz, and the least frequently by the inhabitants of Kuryłówka, but no changes in this respect were observed by the inhabitants of Wydrze. The development of new functions (areas of services and business) was indicated more often by the inhabitants of Wydrze and Bachórz, and the latter definitely more often than others also noticed the construction of solar panels (Table 6).

The assessment of landscape changes as positive varied among the respondents from different localities (Table 7). The inhabitants of Wydrze were most often definitely convinced about the positive changes in the landscape, however some of these inhabitants did not share this opinion. The residents' opinion often differed on this subject, with the same frequency indicating the answers rather yes and rather not. The inhabitants of Zarzecze, Wojtkowa, Chałupki and Kuryłówka were rather convinced of the positive changes – although the opinions of the inhabitants of the last locality were the most diverse.

The opinions of the respondents from different localities varied significantly on the decisions of a commune regarding spatial solutions take into account the spatial order, harmony and beauty of the landscape of their localities (Table 8). This opinion was most often shared by the inhabitants of Chałupki and Kuryłówka, less often by the inhabitants of Wojtkowa and Wydrza, and the least frequently by the inhabitants of Bachórz and Zarzecze.

The respondents were most often not afraid of changes threatening the quality of the landscape in their place of residence, such as the disappearance of regional architecture, lack of care for the quality of green areas, environmental pollution (garbage, exhaust fumes, dirty river), wind farms, glaring colours of building facades, or changes to the rural landscape caused by pressure on the part of modern construction (Table 9). In this respect, no differences were found between the opinions of the respondents living in different localities. On average, every fourth

**Table 6** Landscape changes in the participants' place of residence over the last twenty years.

| Change | Wydrze | Bachórz | Zarzecze | Wojtkowa | Chałupki | Kuryłówka | Total | $p$ |
|---|---|---|---|---|---|---|---|---|
| New residential development areas | 55.0% | 58.8% | 80.4% | 30.0% | 68.0% | 63.5% | 59.2% | <0.001 |
| Construction of new local roads | 16.7% | 13.7% | 9.6% | 58.0% | 16.0% | 19.2% | 21.9% | <0.001 |
| A clear reduction in the area of green areas | 6.7% | 14.0% | 0.0% | 0.0% | 0.0% | 7.7% | 4.8% | 0.002 |
| A clear increase in the area of green areas | 0.0% | 19.6% | 5.8% | 8.0% | 4.0% | 1.9% | 6.4% | 0.001 |
| Intensive urbanization processes and disappearance of agricultural functions in rural areas | 1.7% | 7.8% | 1.9% | 0.0% | 0.0% | 5.8% | 2.9% | 0.088 |
| The emergence of new functions (service and business areas) | 11.7% | 15.7% | 1.9% | 2.0% | 0.0% | 0.0% | 5.4% | <0.001 |
| Wind farm | 0.0% | 3.9% | 0.0% | 0.0% | 0.0% | 0.0% | 0.6% | 0.064 |
| Solar panels | 8.3% | 74.5% | 1.9% | 2.0% | 12.0% | 0.0% | 16.2% | <0.001 |

**Table 7** Assessment of landscape changes as positive.

| Opinion | Wydrze | Bachórz | Zarzecze | Wojtkowa | Chałupki | Kuryłówka | Total |
|---|---|---|---|---|---|---|---|
| Definitely yes | 61.7% | 9.8% | 0.0% | 30.0% | 26.0% | 34.6% | 27.9% |
| Rather yes | 10.0% | 45.1% | 75.0% | 54.0% | 58.0% | 42.3% | 46.4% |
| Rather no | 28.3% | 45.1% | 15.4% | 8.0% | 0.0% | 9.6% | 18.1% |
| Definitely no | 0.0% | 0.0% | 0.0% | 2.0% | 0.0% | 1.9% | 0.6% |
| I don't know/ hard to say | 0.0% | 0.0% | 9.6% | 6.0% | 16.0% | 11.5% | 7.0% |
| Total | 100.0% | 100.0% | 100.0% | 100.0% | 100.0% | 100.0% | 100.0% |
| $p$ | $\chi^2(20)=132.59$, $p<0.001$ <br> $\chi^2NW(20)=159{,}12$, $p<0{,}001$ | | | | | | |

**Table 8** Respondents' opinions regarding if the decisions of the commune regarding spatial solutions take into account the spatial order, harmony and beauty of the landscape in the place of their residence.

| Opinion | Wydrze | Bachórz | Zarzecze | Wojtkowa | Chałupki | Kuryłówka | Total |
|---|---|---|---|---|---|---|---|
| Yes | 61.7% | 45.1% | 40.4% | 68.0% | 80.0% | 78.9% | 62.2% |
| No | 10.0% | 27.5% | 15.4% | 18.0% | 2.0% | 5.8% | 13.0% |
| I don't know/ hard to say | 28.3% | 27.5% | 44.2% | 14.0% | 18.0% | 15.4% | 24.8% |
| Total | 100.0% | 100.0% | 100.0% | 100.0% | 100.0% | 100.0% | 100.0% |
| *p* | $\chi^2(10)=41.56$, $p<0.001$ <br> $\chi^2NW(10)=42{,}36$, $p<0{,}001$ | | | | | | |

**Table 9** Respondents' opinions on the elements that threaten the quality of the landscape in their localities.

| Element | Wydrze | Bachórz | Zarzecze | Wojtkowa | Chałupki | Kuryłówka | Total | *p* |
|---|---|---|---|---|---|---|---|---|
| Spatial chaos | 5.0% | 9.8% | 13.5% | 12.0% | 34.0% | 11.5% | 14.0% | 0.001 |
| Loss of identity | 3.3% | 3.9% | 17.3% | 8.0% | 4.0% | 5.8% | 7.0% | 0.044 |
| Natural impoverishment of the landscape | 6.7% | 5.9% | 7.7% | 0.0% | 2.0% | 15.4% | 6.4% | 0.031 |
| Disappearance of regional architecture | 1.7% | 7.8% | 7.8% | 4.0% | 12.0% | 13.5% | 7.6% | 0.166 |
| Lack of care for the aesthetics of private facilities | 5.0% | 23.5% | 15.4% | 6.0% | 4.0% | 15.4% | 11.4% | 0.008 |
| Lack of care for the quality of green areas | 6.7% | 15.7% | 11.5% | 4.0% | 2.0% | 5.8% | 7.6% | 0.096 |
| Environmental pollution (garbage, exhaust fumes, polluted river) | 5.0% | 7.8% | 5.8% | 14.0% | 18.0% | 9.6% | 9.8% | 0.185 |
| Visual aggression of advertisements / signs | 1.7% | 9.8% | 1.9% | 0.0% | 0.0% | 7.7% | 3.5% | 0.020 |
| Wind farms | 1.7% | 2.0% | 0.0% | 0.0% | 0.0% | 0.0% | 0.6% | 0.587 |
| Solar panels | 0.0% | 74.5% | 0.0% | 2.0% | 0.0% | 1.9% | 12.7% | <0.001 |
| Lack of aesthetics of public facilities | 0.0% | 7.8% | 1.9% | 0.0% | 2.0% | 0.0% | 1.9% | 0.025 |
| The glaring colours of the facades of buildings | 0.0% | 3.9% | 1.9% | 0.0% | 0.0% | 0.0% | 1.0% | 0.197 |
| Changes in the rural landscape due to pressure from modern construction | 0.0% | 2.0% | 0.0% | 0.0% | 2.0% | 2.0% | 1.0% | 0.668 |
| Lack of aesthetics of small architecture elements (e.g. bus stops, fences, benches) | 0.0% | 43.1% | 0.0% | 0.0% | 0.0% | 0.0% | 7.0% | <0,001 |
| Other items | 20.0% | 0.0% | 0.0% | 0.0% | 0.0% | 3.9% | 4.4% | <0.001 |
| Lack of threats | 45.0% | 7.8% | 15.4% | 48.0% | 18.0% | 9.6% | 24.4% | <0.001 |

respondent did not notice any threats. The inhabitants of Wydrze and Wojtkowa most often pointed to the lack of any threats. This relationship was statistically significant ($p$ <0.001). Concerns related to the remaining elements that threaten the quality of the landscape were different in the six groups ($p$ <0.05). Spatial chaos was most often feared by the inhabitants of Chałupki, the disappearance of identity – by the inhabitants of Zarzecze, the impoverishment of the natural landscape – by the inhabitants of Kuryłówka, the lack of care for the aesthetics of private buildings – by the inhabitants of Bachórz, Zarzecze and Kuryłówka, the visual aggression of advertisements / signs – by the inhabitants of Bachórz and Kuryłówka, solar panels, the aesthetics of objects and the aesthetics of small architecture elements were most often feared by the inhabitants of Bachórz, while the inhabitants of Wydrze were most often afraid of other elements.

The assessment of small architecture in terms of

**Table 10** Land cover categories within the studied villages in relation to the Corine Land Cover category (Numbers in the 3rd column (112, 211, 231, ..., etc.) are CORINE Land Cover Codes).

| Level 1 | Level 2 | Level 3 | |
|---|---|---|---|
| Artificial surfaces | Urban fabric | 112 | Discontinuous urban fabric |
| | Industrial, commercial and transport units | 121 | Industrial or commercial units |
| | | 122 | Road and rail networks and associated land |
| Agricultural areas | Arable land | 211 | Non-irrigated arable land |
| | Meadows and pastures | 231 | Meadows, pastures |
| | Heterogeneous agricultural areas | 242 | Complex cultivation patterns |
| | | 243 | Land principally occupied by agriculture, with significant areas of natural vegetation |
| Forests and semi-natural ecosystems | Forests | 311 | Deciduous forests |
| | | 312 | Coniferous forests |
| | | 313 | Mixed forests |
| | | 324 | Forests and shrub vegetation in a state of change |
| Water bodies | Inland wetlands | 511 | Watercourse |

having a uniform appearance differed in the opinions of the inhabitants of the six examined localities ($p < 0.001$). The inhabitants of Chałupki were the least likely to mention a uniform appearance, the inhabitants of Bachórz – rarely and the inhabitants of Kuryłówka – relatively rarely. In this respect, it was assessed best by the inhabitants of Wojtkowa, and relatively well by the inhabitants of Wydrze and Zarzecze.

The analysis of the variability of the spatial structure of the landscape on the basis of CORINE Cover Land data from 2000 and 2018, changes took place within a 2.5 km radius around the 6 surveyed towns become noticeable. Based on the data from the CLC 2000 and 2018 database, data was obtained on the areas occupied by the separate land cover categories. The authors reduced its usefulness to the analysis of the landscape structure of selected types of use in the studied sub-mountain villages. There were 12 land cover classes within the studied areas, out of 31 in Poland (Table 10). The obtained results of measures and indicators are presented in Table 11.

The analysis of CORINE Land Cover maps allowed for the determination of the dominant types of use in the vicinity of the analyzed towns. Bachórz, Chałupki, Kuryłówka and Zarzecze are locations with a predominance of arable land (arable land, meadows and pastures). On the other hand, Wydrze and Wojtkowa are located in the vicinity of large forest complexes (Table 11). The localities also differed in the area of buildings, Zarzecze and Kuryłówka are characterized by their highest share, while only the complex systems of cultivation and plots, characteristic of small villages, were found in Wojtkowa. In the years 2000 – 2018, changes in land use were observed in the vicinity of all the surveyed towns in the years 2000 – 2018.

The analysis showed the appearance of loose housing in the villages of Bachórz and Wydrze and its significant increase in Chałupki and Kuryłówka. In 2000, rural buildings in Bachórz and Wydrze were classified into complex systems of cultivation and plots. The percentage increase in afforestation is noticeable in the villages of Bachórz and Kuryłówka, while a slight upward trend in this type of use could be observed in the case of Wojtkowa and Chałupki. A clear increase in the area of agricultural land took place only in the village of Chałupki (Fig. 2 and Table 11).

The expert analysis also indicated the lack of planning documents for individual towns, while the on-site inspection revealed the deficiencies of the landscape in the form of aggressive advertisements, construction inadequate for the rural landscape, or dispersed settlement.

## 5 Discussion

The study presented the attractiveness of the landscape from the perspective of the inhabitants of Subcarpathian villages and compared them with the results of the expert approach. A specific dualism is visible in the thesis – the actions of planners and social evaluation. This dual nature of the phenomenon of landscape perception was assessed in the work from the point of view of science and theory, and from the point of view of the recipient, inhabitant and user. The correlation between the perceived and formally assessed value of a landscape has been compiled to better understand the similarities and differences between them. Experts' assessments of landscapes differ from the local residents.

**Table 11** Area of selected types of land use and the percentage of its increase or decrease in the period 2000-2018 (Numbers in the in the second column (112, 211, 231, ..., etc.) are CORINE Land Cover Codes)

| Village | CORINE Land Cover Codes | Area (ha) in 2000 | Area (ha) in 2018 | Increase or decrease (%) |
|---|---|---|---|---|
| Bachórz | 112 Discontinuous urban fabric | - | 113.1 | |
| | 211 Non-irrigated arable land | 1079.2 | 1121.1 | 3.9 |
| | 231 Pastures | 2.8 | 2.8 | 0.0 |
| | 242 Complex cultivation patterns | 256.3 | 100.6 | -60.7 |
| | 243 Land principally occupied by agriculture, with significant areas of natural vegetation | 358.7 | 184.5 | -48.6 |
| | 311 Deciduous forest | 108.1 | 164.3 | 52.0 |
| | 312 Coniferous forest | 46.6 | 28.8 | -38.2 |
| | 313 Mixed forest | 37.2 | 178.1 | 378.3 |
| | 511 Water courses | 74.5 | 70.1 | -5.9 |
| Chałupki | 112 Discontinuous urban fabric | 35.4 | 166.9 | 371.3 |
| | 121 Industrial or commercial units | 25.0 | 36.5 | 45.7 |
| | 122 Road and rail networks and associated land | 17.3 | 74.9 | 332.4 |
| | 211 Non-irrigated arable land | 881.1 | 1056.5 | 19.9 |
| | 231 Pastures | 679.6 | 431.8 | -36.5 |
| | 242 Complex cultivation patterns | 164.0 | - | -100.0 |
| | 312 Coniferous forest | 3.3 | 3.3 | 0.0 |
| | 313 Mixed forest | 157.6 | 166.3 | 5.5 |
| | 324 Transitional woodland-shrub | 0.0 | 26.8 | - |
| Kuryłówka | 112 Discontinuous urban fabric | 148.2 | 293.5 | 98.1 |
| | 211 Non-irrigated arable land | 1085.9 | 1035.9 | -4.6 |
| | 231 Pastures | 236.3 | 230.9 | -2.3 |
| | 242 Complex cultivation patterns | 178.7 | 1.4 | -99.2 |
| | 243 Land principally occupied by agriculture, with significant areas of natural vegetation | 70.3 | 67.3 | -4.3 |
| | 311 Deciduous forest | 9.4 | 9.4 | 0.0 |
| | 312 Coniferous forest | 147.1 | 237.4 | 61.3 |
| | 511 Water courses | 87.4 | 87.4 | 0.0 |
| Wojtkowa | 211 Non-irrigated arable land | 454.7 | 390.5 | -14.1 |
| | 231 Pastures | 168.4 | 139.8 | -17.0 |
| | 242 Complex cultivation patterns | 224.3 | 218.0 | -2.8 |
| | 243 Land principally occupied by agriculture, with significant areas of natural vegetation | 30.1 | 95.7 | 217.7 |
| | 311 Deciduous forest | 121.3 | 96.6 | -20.3 |
| | 312 Coniferous forest | 894.5 | 942.4 | 5.4 |
| | 313 Mixed forest | 70.1 | 76.8 | 9.5 |
| | 324 Transitional woodland-shrub | - | 3.1 | - |
| Wojtkowa | 112 Discontinuous urban fabric | - | 98.1 | - |
| | 211 Non-irrigated arable land | 573.5 | 454.2 | -20.8 |
| | 231 Pastures | 71.6 | 40.1 | -44.0 |
| | 242 Complex cultivation patterns | 194.7 | 115.0 | -41.0 |
| | 243 Land principally occupied by agriculture, with significant areas of natural vegetation | 100.0 | 6.8 | -93.2 |
| | 311 Deciduous forest | 46.5 | 46.5 | 0.0 |
| | 312 Coniferous forest | 426.7 | 491.6 | 15.2 |
| | 313 Mixed forest | 550.4 | 645.4 | 17.3 |
| | 324 Transitional woodland-shrub | - | 65.9 | - |
| Zarzecze | 112 Discontinuous urban fabric | 488.9 | 527.3 | 7.9 |
| | 211 Non-irrigated arable land | 1009.6 | 1104.1 | 9.4 |
| | 231 Pastures | 53.0 | 54.9 | 3.7 |
| | 242 Complex cultivation patterns | 105.9 | 45.0 | -57.5 |
| | 243 Land principally occupied by agriculture, with significant areas of natural vegetation | 246.8 | 100.0 | -59.5 |
| | 313 Mixed forest | 59.3 | 129.9 | 119.3 |
| | 324 Transitional woodland-shrub | - | 2.7 | - |

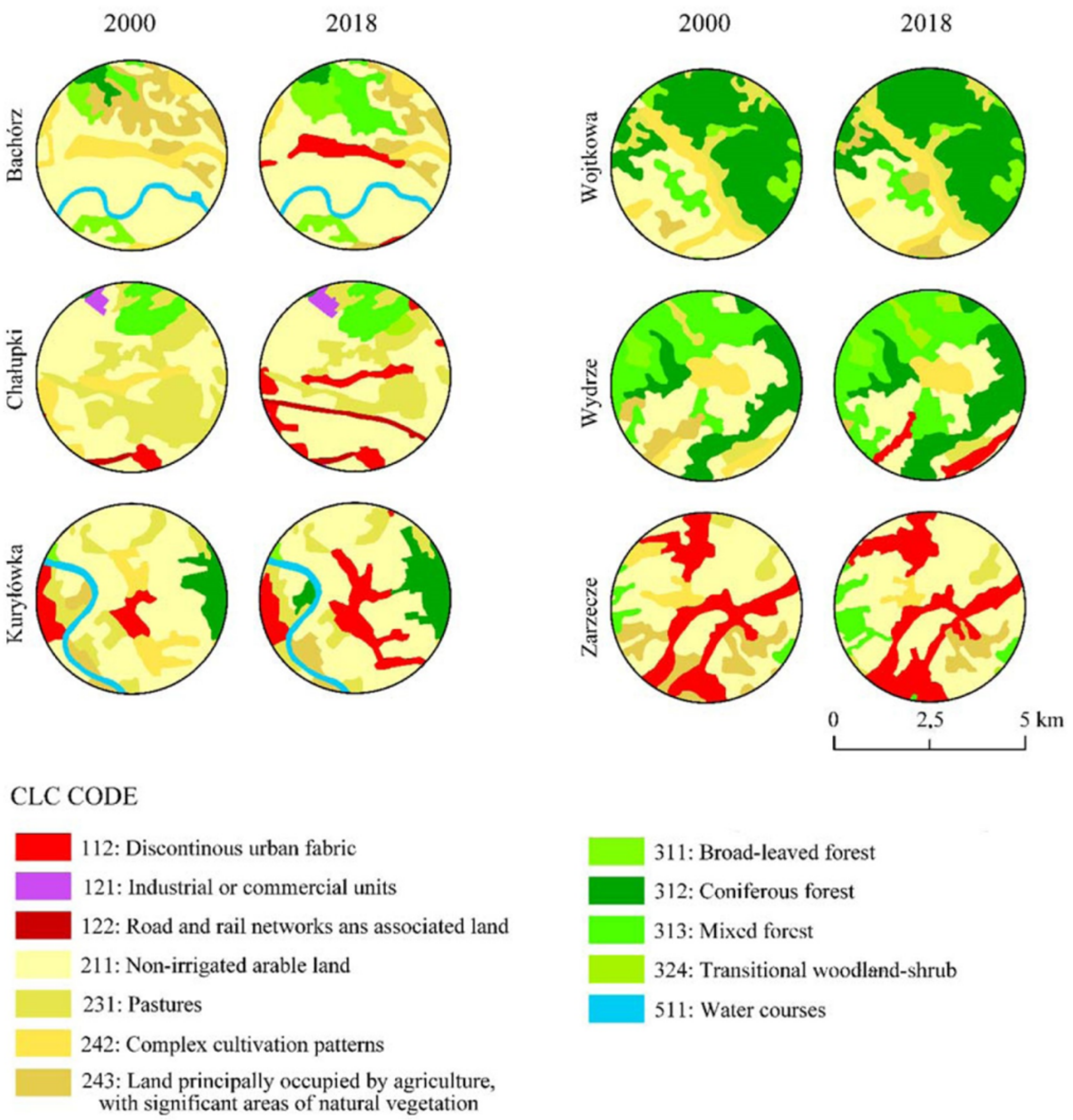

**Fig. 2** Analysis of the variability of the spatial structure of the landscape on the basis of CORINE from 2000 and 2018.

Experts evaluate landscapes visually, while residents often give higher value to local landscapes to which they have developed a sense of belonging. This is also confirmed by the claim made by Wartmann et al. (2021).

Inhabitants of the studied Subcarpathian villages perceive the place they live in rather as attractive in terms of landscape. The results are in line with Wartmann et al. (2021) who note that openness of vision is positively related to visual quality assessments, which refers to the nature of rural landscapes. Our discovery also corresponds to the suggestions of Ridding et al. (2018), where rural landscapes are perceived as an environment for living, well-being, as well as for the identification of a given place.

The results indicate that the village of Wojtkowa, located the farthest from both the small (Ustrzyki Dolne) and the large urban centre (Rzeszów), was considered the most attractive in terms of landscape. These results are in line with Bielewska (2011), indicating that the Carpathian region is currently undergoing intensive spatial and landscape transformations. As a result, valuable natural areas are absorbed. The villages of the Carpathian region lose their historical compositional legibility, plunging into planning chaos caused by i.e. through the modern dispersed residential areas. The inhabitants of Zarzecze, a town located in the immediate vicinity of a small (Boguchwała) and large urban centre (Rzeszów), are most worried about this situation. This Carpathian village is particularly exposed to intense urbanization and the loss of valuable geographic, natural, and cultural and historical sites. The investment pressure in this mountain region means that new forms of development are constructed in the open landscape areas. According to Hajduk (2018), research on the spatial management system based on the example of 18 Polish provincial cities indicate that the highest investment pressure and, at the same time, poor planning coverage, occurs in the capital of the Podkarpackie province – Rzeszów.

The respondents notice the changes in the landscape taking place in the inhabited locality over the last twenty years. These changes (new residential areas, construction of new local roads, a marked reduction in the area of green areas, a marked increase in the area of arranged green areas, intensive urbanization processes and the disappearance of agricultural functions in rural areas, the emergence of new functions, wind farm, solar panels) are perceived positively. We conclude that landscapes perceived as valuable are often described by the respondents as places of a unique nature that are intended for different users or for different activities. This is in line with the findings of Hermes et al. (2018), Rewitzer et al. (2017) that landscapes are important for people as a living environment, as well as for individuals' relationships with specific places.

The respondents are convinced that the commune's decisions regarding spatial solutions take into account the spatial order, harmony and beauty of the landscape in the place of their residence. Meanwhile, none of the examined towns has a local village spatial development plan – i.e. an act of local law specifying the purpose, conditions of land development and development, and the location of public purpose investments. Scientific studies (Berziński 2013; Niedziałkowska & Beunenb 2019; Gorzym-Wilkowski & Trykacz 2022) indicate that in the absence of a local plan, there is no effective tool for spatial coordination, and thus areas are not protected against disharmonious development. The decisions of municipal governments focus on the implementation of development plans aimed at securing voters. They usually concern the designation of new investment areas, their development and road construction. Such activities are often accompanied by an ecological imbalance or degradation of cultural heritage, but they are always associated with visual changes that are usually not perceived by the local community as dangerous (Górka 2018). Angel and Pietrzak (2009) believe that the transformations of the countryside currently taking place make it impossible to have any illusions – the harmonious cultural landscape of a traditional village is being changed as a result of changes in the spatial arrangement and progressive urbanization. The study indicated that the landscape changes were most often noticed by the inhabitants of Kuryłówka, the village with the highest demographic growth in the recent time. As noted by Maik (2009), the degree of demographic changes in the Carpathian region increases with the progress of its urbanization.

According to Bogdanowski (1998), cultural landscapes are an expression of human economic activity. Any revalorization of space and landscape should be shaped by society aware of local values. The awareness of people living in a given rural landscape should be shaped by the knowledge shared, promoted and supported by research. The media and industry policies currently play an important role in popularizing the right attitudes towards the landscape (Górka 2018). The current increase in urbanization, noticed by experts in the GIS environmental study, and little involvement in the protection of the environment, proves the lack of public awareness in this field. This is due to the changes that took place in Polish rural areas and agricultural activity at the turn of the 20th and 21st centuries, which were the result of the political transformation. In terms of land use, the area of fallow land has increased. There have been changes in the level of development, farm size and production directions. After Poland joined the European Union (2003), certain regions saw an increase in the dynamics of investments in agriculture and transformations of the functional structure. In areas with high natural and cultural values, a development of rural tourism and agritourism has taken place (Baran-Zglobicka 2014). The centuries-old cultural heritage of rural landscapes has undergone significant destruction during the period of systemic changes (Deslatte et al. 2022), when the city was created as a place optimal for life and personal and social development (Tratsela et al. 2012; Gajdek 2018). A valuable initiative in the field of protection and restoration of the value of the rural landscape is the Rural Renewal Program, originating from Western Europe and its history dates back to the 1970s. In Poland, the Rural Renewal Program has found fertile ground for development in the Opole region, where it has been working very well since 1997. Following the example of this region, more and more villages are joining the program, incl. Pomeranian and Silesian. The idea of the Rural Renewal Program is to activate the inhabitants, interest in their own village and the broadly understood identity of the place, tradition and culture, while ensuring all the achievements of modern technology (Idziak & Wilczyński 2013). In the process of revitalizing a rural landscape and carrying out effective activities in the field of its protection, it is important to determine the features that distinguish a given village from other localities (Niedźwiecka-Filipiak 2018). This is the case with the concept of the Network of the Most Beautiful Villages, created and developed in France, and also popularized in other parts of the world, including some provinces in Poland (Networks of the Most Beautiful Villages – expert study of the project, Opole 2012). An action promoting good architectural forms, continuing local traditions, is the organization of competitions in which solutions are selected (and sometimes also implemented) which are an example of good practices in shaping contemporary construction, which can combine new technologies, comfort and uniqueness with the regional construction tradition (Rumińska 2013).

The beginning of the 21st century brought many new challenges, also in the spatial dimension, such as the COVID-19 pandemic and the war conflict in Ukraine. This situation provokes i.e. to reflect on the quality of the environment in which we live, and this indirectly relates to the features of the landscape that are co-created by local communities. The harmony of the landscape does not mean only superficial beauty expressed only in providing it with relatively aesthetic elements. Harmoniousness is the result of the proper functioning of the individual components of the landscape, understood as a multi-faceted system that should be changed and shaped in a responsible and conscious manner (Luc 2018), as gives Tengberg et al. (2012) designing landscape is not a return to the past, but drawing conclusions and awareness of our impact on the landscape.

## 6 Conclusion

The rural landscapes of the Carpathian region in Poland are undergoing significant transformations that threaten the protection of key values related to the natural values and cultural heritage of the surveyed towns. This is a common problem in many of Europe's countryside. Considerations about the landscape, especially the rural landscape (in terms of visual attractiveness) are not obvious for the average rural inhabitant, who treats economic and technological development as superior values, also in the field of landscape management. There is a need for multifaceted actions that will enable effective protection of the rural cultural landscape, including:

1. Active inclusion of landscape architects in the process of planning rural areas. Landscape architects play a special role in shaping and promoting a harmonious landscape. Thanks to the comprehensive education process, taking into account both environmental and cultural aspects, their task is to cultivate among the local community the need to maintain the individual character of a given town against the background of the surrounding area, country or continent.

2. Protection of cultural and natural heritage in accordance with the needs of the present. Well-functioning village is not a kind of museum where no changes should be made. In order to be attractive, the rural landscape must also develop and modernize. Revitalization processes should primarily concern giving old forms new functions, corresponding to a given socio-economic situation, but at the same time preserving their rural character. This will help to stop the negative tendency of rural areas attempting to resemble urban.

3. Promoting a visually harmonious landscape. Visualizing a harmonious rural landscape supports the process of creating collective imagination, which in turn shapes spatial reality. Landscape visualization tools, such as drawings, photos, maps, paintings and films, play an important role in the process of participatory planning or public consultation procedures. An image, an attractive, memorable visualization, over time shapes the correct perception of everyday surroundings.

4. Raising the awareness of rural residents of objective benefits (ecological, economic and social) resulting from the protection of local traditions and landscape. Thanks to the protection of the cultural environment, but also its natural ecosystems, the richness of the regional diversity can be preserved in the landscape, which allows the development of rural tourism and the promotion of the rural landscape.

5. Organize local activating actions or artistic events dealing with the above-mentioned topics.

## Open Access

This article is licensed under a Creative Commons Attribution 4.0 International License, which permits use, sharing, adaptation, distribution and reproduction in any medium or format, as long as you give appropriate credit to the original author(s) and the source, provide a link to the Creative Commons license, and indicate if changes were made. The images or other third party material in this article are included in the article's Creative Commons license, unless indicated otherwise in a credit line to the material. If material is not included in the article's Creative Commons license and your intended use is not permitted by statutory regulation or exceeds the permitted use, you will need to obtain permission directly from the copyright holder. To view a copy of this license, visit http://creativecommons.org/licenses/by/4.0/.
